# Prevalence of Depression in Patients with Chronic Kidney Disease Stage 5 on Hemodialysis at a Tertiary Care Center

**DOI:** 10.31729/jnma.4408

**Published:** 2019-06-30

**Authors:** Krishna Kumar Agrawaal, Pramod Kumar Chhetri, Pradip Man Singh, Dhiraj Narayan Manandhar, Prakash Poudel, Anjeel Chhetri

**Affiliations:** 1 Department of Nephrology, Nepal Medical College, Kathmandu, Nepal; 2 Department of Psychiatry, Nepal Medical College, Kathmandu, Nepal

**Keywords:** *depression*, *hemodialysis*, *Nepal.*

## Abstract

**Introduction:**

Co-morbid depression impacts negatively on quality of life in Chronic Kidney Disease patients. This study was done to calculate prevalence of depression in patients on hemodialysis (HD) using Patient Health Questionnaire-9, Hamilton Rating Scale for Depression-17 and International Classification of Disease-10 classification of mental and behavioural disorders.

**Methods:**

It was descriptive cross-sectional study conducted from November 2017 till June 2018. Ethical approval was taken from Ethical Review Board, Nepal Health Research Council. Informed and written consent was taken. Patients undergoing hemodialysis at Nepal Medical College for more than 3 months duration were included in study. Patients on hemodialysis were asked to fill validated Nepali translated version of Patient Health Questionnaire-9. Psychiatrist administered Max Hamilton Rating scale for diagnosis and categorization of depression and confirmed depression based on International Classification of Disease-10. Statistical Package for Social Sciences version 20 was used for statistical analysis.

**Results:**

The study was done among 100 patients. Prevalence of depression was 78 (78%) using Patient Health Questionnaire-9 and 65 (65%) using Max Hamilton Rating scale and 51 (51%) using International Classification of Disease -10. Mean depression in males using Patient Health Questionnaire -9 was 7±4.33 and in females was 11.04±5.90. Median age was 47.5 years. The most common symptom was fatigue among 82 (82%).

**Conclusions:**

There is a high prevalence of depression in patients with Chronic Kidney Disease stage 5 on hemodialysis compared to general population.

## INTRODUCTION

Depression is the most common psychiatric problem in Chronic Kidney disease (CKD) patients. Co-morbid depression impacts negatively on quality of life in CKD.^1^

It is unclear if self-reported depression rating scales can be used accurately. There are different scales used in dialysis patients like Beck Depression Inventory (BDI) and Quick Inventory of Depression Symptomatology (QIDS-SR).^2,3^ In meta-analysis by Stripolli et al. it was found that using self- or clinician-administered rating scales, prevalence of depressive symptoms for CKD stage 5 was higher than other stages.^4^ The BDI, Hamilton Rating Scale for Depression (HAD-17), Nine-Question Patient Health Questionnaire (PHQ-9) are some of measures that have been used to screen for depression in patients with end-stage renal disease (ESRD).^5^ In study conducted in Nepal, PHQ-9 was validated for its use in depression. PHQ-9 is preferred tool for use in low and middle income countries (LMICs).^6^

This study was done to calculate prevalence of depression in patients on hemodialysis (HD) using PHQ-9, HAD-17 and ICD-10 classification of mental and behavioural disorders.

## METHODS

This descriptive cross-sectional study was conducted at Nepal Medical College, Kathmandu from November 2017 till June 2018. Ethical approval was taken from Ethical Review Board, Nepal Health Research Council. Sample size was calculated using the sample size calculation formula:


$$\text{n}={\text{Z}}^{2}(\text{p}\times \text{q)/d2}$$

where,

n = required sample size
Z = 1.96 at 95% Confidence Interval
P = estimated prevalence of ESRD population (5% taking an account of increase in prevalence from previous studies)
q = 1-p
d = allowable error, 5%

Based on the above formula the minimum estimated sample size at 95% confidence interval and 5% error, total sample size calculated was 73. Taking non-response rate of 10%, the calculate sample size is 80. We took 100 patients in the study. Patients above the age of 18 years, those giving informed written consent to be enrolled in study and on HD at Nepal Medical College more than 3 months duration were included in the study. Patients on HD were asked to fill the Nepali translated version of PHQ-9 questionnaire, which is already validated tool for screening and diagnosis of depression. The patients took approximately one minute to fill the PHQ-9 and were not allowed to cross talk. The psychiatrist administered Max Hamilton Rating scale for the diagnosis and categorization of depression. Psychiatrist also confirmed the depression diagnosis with personal interview with each patient in the dialysis unit itself and labelled them based on ICD-10 classification of mental and behavioural disorders (Diagnostic criteria for research). SPSS v. 20 was used for statistical analysis.

## RESULTS

Total number of patients included in final analysis were 100. Male: Female ratio was 1.21:1 (Table 1).

**Table 1.** Showing baseline characteristics of patients (n=100).

Characteristics	Categories	n (%)
Age	<20 years	1 (1)
	20-39 years	35 (35)
	40-59 years	36 (36)
	≥60 years	28 (28)
Median age in years (IQR)		47.5 (33.0 – 60.75)
Duration since dialysis	3-6 months	9 (9)
	7-12 months	22 (22)
	13-24 months	33 (33)
	25-48 months	20 (20)
	>48 months	16 (16)
Median duration since HD in months (IQR)		24.0 (9.25-36.00)
PHQ-9 Total scores	<10	63 (63)
	≥10	37 (37)
	≥15	16 (16)
	≥20	5 (5)

Mean PHQ-9 score and HAD-17 score was 8.86±5.48 and 12.33± 7.01 respectively.

Most common symptom was fatigue 82 (82%) based on PHQ-9 (Table 2).

**Table 2.** Showing frequencies of different symptoms of PHQ-9 (n=100).

Characterstics	n (%)
Anhedonia	65 (65)
Depressed mood	67 (67)
Sleeping difficulties	56 (56)
Fatigue	82 (82)
Appetite problems	61 (61)
Blaming oneself	53 (53)
Concentrating difficulties	46 (46)
Psychomotor agitation	82 (82)
Suicidal ideation	45 (45)

Grading of depression based on PHQ-9 and HAD-17 and ICD-10 (Table 3).

**Table 3.** Level of Depression using different scales (n = 100).

Level of Depression	Grading	n (%)
Based on PHQ-9	Minimal	22 (22)
	Mild	41 (41)
	Moderate	21 (21)
	Moderately	11 (11)
	Severe	
	Severe	5 (5)
based on Max	Normal	35 (35)
Hamilton score	Mild	22 (22)
(HAD-17)	Moderate	18 (18)
	Severe	21 (21)
	Very Severe	4 (4)
based on ICD-10	No Depression	49 (49)
	Mild Depressive	13 (13)
	Episode without	
	Somatic	
	Syndrome	
	Mild Depressive	2 (2)
	Episode with	
	Somatic	
	Syndrome	
	Moderate	7 (7)
	Depressive	
	Episode without	
	Somatic	
	Syndrome	
	Moderate	13 (13)
	Depressive	
	Episode with	
	Somatic	
	Syndrome	
	Severe	16 (16)
	Depressive	
	episode without	
	psychotic	
	symptoms	

Prevalence of depression using PHQ-9, HAD-17 and ICD-10 was 78%, 65% and 51% respectively. Mean depression in males using PHQ-9 was 7±4.33 and in females was 11.04±5.90. Similarly, the mean depression in males using HAD-17 score was 11.76±6.75 and in females was 13.00±7.32 with CI ranging from 1.55 to 4.03.

## DISCUSSION

In this series of 100 patients on HD at Nepal Medical College, we enrolled patients who were in Nepal Governments' Bipanna Program i.e. free HD services. We excluded patients who received HD out of pocket to eliminate the economic confounding factor leading to depression. The median age of patients was 47 years which is similar to study in BPKIHS and previous study at NMCTH.^7,8^

In our study, age had no impact on depression as there was no difference in means. Women had more instances of depression which was comparable to other studies.^9,10^ The median duration of dialysis patients in this study was 24 months.

In a study done by Khaled et al, found that fatigue was the most common symptoms in patients with dialysis and this was similar in our study. They also compared PHQ-9 with 30 item dialysis symptom index and found that a median of PHQ-9 score of more than 9 is comparable.^11^

The mean PHQ-9 score in our study was 9 which is comparable to other studies. Here we used HAD-17 score which is considered as a standard for depression screening. No direct comparison has been done till date in ESRD population but in a study by Williams et al. in Parkinson patient with depression they compared three clinician led scales.^12^

Overall prevalence of depression in our series was 78% using PHQ-9 score and the 65% using HAD-17 scores. Around 16% of the patients had severe depression in patients using the PHQ-9 and 25% had severe depression using the HAD-17 scores. The patients' self administered the proforma for PHQ-9 but HAD-17 was filled by the psychiatrist and final diagnosis was made by the psychiatrist himself. This might have led to exclusion of patient who had mild depression on PHQ-9 scale. Similarly the HAD-17 score also labelled most of the patients as no depression who were having mild depression on PHQ-9. This may have caused the difference in the prevalence of depression using the different tools.

The advantage of PHQ-9 was that it was self administered and took approximately a minute to fill by the patient but biggest disadvantage was its low specificity which was similar in other studies.^13,14^

Based on the above presented results and comparing other studies with PHQ-9, it is found that the scale is having good sensitivity and due to its easy administration, it can be used to screen the patients but once the score of PHQ-9 is >9 the patients need to be subjected for psychiatric evaluation. The depression adds to morbidity and mortality to patients hence this cost effective tool can be used.

## CONCLUSIONS

There is a high prevalence of depression in patients on hemodialysis compared to general population. Patients with score >9 on PHQ-9 can be subjected to HAD-17 scale and if facilities available diagnosis should be confirmed by psychiatrist.
